# Safety of fluralaner, a novel systemic antiparasitic drug, in MDR1(-/-) Collies after oral administration

**DOI:** 10.1186/1756-3305-7-86

**Published:** 2014-03-06

**Authors:** Feli M Walther, Allan J Paul, Mark J Allan, Rainer KA Roepke, Martin C Nuernberger

**Affiliations:** 1 MSD Animal Health Innovation GmbH, Zur Propstei, 55270 Schwabenheim, Germany; 2 University of Illinois College of Veterinary Medicine, 2001 S. Lincoln Ave, Urbana, IL 61802, USA

**Keywords:** Fluralaner, Bravecto™, Dog, Safety, MDR1

## Abstract

**Background:**

Fluralaner is a novel systemic ectoparasiticide for dogs providing long-acting flea- and tick-control after a single oral dose. This study investigated the safety of oral administration of fluralaner at 3 times the highest expected clinical dose to Multi Drug Resistance Protein 1 (MDR1(-/-)) gene defect Collies.

**Methods:**

Sixteen Collies homozygous for the MDR1 deletion mutation were included in the study. Eight Collies received fluralaner chewable tablets once at a dose of 168 mg/kg; eight sham dosed Collies served as controls. All Collies were clinically observed until 28 days following treatment.

**Results:**

No adverse events were observed subsequent to fluralaner treatment of MDR1(-/-) Collies at three times the highest expected clinical dose.

**Conclusions:**

Fluralaner chewable tablets are well tolerated in MDR1(-/-) Collies following oral administration.

## Background

Fluralaner is a novel systemically administered insecticidal and acaricidal product that provides long acting efficacy after oral administration to dogs. Fluralaner belongs to a new class of compounds, the isoxazolines. These compounds have activity against γ-aminobutyric acid- (GABA-) and glutamate-gated chloride channels with significant selectivity for insect neurons over mammalian neurons [1]. A field study has shown that a single fluralaner dose administered orally to dogs provides at least twelve weeks of flea- and tick-control [2]. The long duration of activity offers a more convenient treatment over monthly flea and tick control treatments with a potential compliance advantage, reducing the risk of vector-transmitted diseases.

This systemic treatment will likely be administered to dogs carrying a deletion mutation of the Multi-Drug Resistance gene (MDR1). Dogs homozygous for this mutation do not express functional P-glycoprotein [3], a drug efflux pump highly expressed at the blood–brain barrier that restricts drug accumulation in the central nervous system through an efflux-based transport mechanism [4]. MDR1 mutations have been detected in various dog breeds such as rough- and smooth-coated Collie, Shetland Sheepdog, Australian Shepherd, McNab, Longhaired Whippet, Silken Windhound, Old English Sheepdog, English Shepherd, Border Collie and Wäller [5,6]. Homozygous mutations (MDR1(-/-)) are associated with an increased risk of neurotoxicity for multiple drugs, leading to clinical findings such as depression, mydriasis, salivation, tremor, ataxia and coma [3,7-10]. Although MDR1(-/-) dogs do not express a functional P-glycoprotein, inter-individual variability in sensitivity is reported [7,11]. In addition to the low *in vitro* affinity of fluralaner to mammalian neuronal receptors [1], fluralaner did not cause any signs of neurotoxicity in a previous high-dose safety study in healthy dogs [12] nor in a field study [2]; this study was designed to confirm the safety of oral fluralaner administration in dogs with proven MDR1(-/-) mutation. To account for variability in susceptibility, fluralaner was administered at overdoses, i.e. 3 times the highest expected clinical dose of 56 mg/kg BW [2].

## Methods

This single-center, randomized, parallel-group and investigator-blinded study included sixteen healthy male and female rough-coated Collies, 1.7 - 4.0 years of age (mean 1.9 years) and weighing 16–27 kg (mean 22 kg). Dogs were from four different litters, with three different litters per study group. All Collies were homozygous for the MDR1 deletion mutation, as confirmed by gene testing (http://www.vetmed.wsu.edu/VCPL).

This study was conducted in Michigan, USA, in compliance with the Animal Welfare Act as overseen by the United States Department of Agriculture (USDA) and ethical approval was obtained before the start of the study. Study was approved by the Institutional Animal Care and Use Committee (IACUC no. CHK-13-0419).

Healthy Collies, based on initial physical and clinical pathological evaluation, were housed individually and fed a standard commercial diet at recommended rates. Collies were randomly allocated to study groups using the following block randomization procedure: dogs were separated by gender and ranked in ascending order of body weight; if two dogs had identical bodyweights, they were sub-ranked by increasing microchip number. The top two dogs within each gender formed a block that was randomly allocated between each of the two study groups, and the process repeated until 4 male and 4 female dogs were allocated to each study group. A total of 8 Collies received fluralaner and 8 sham dosed Collies served as controls.

The expected dose range for fluralaner administration during routine clinical use is between 25 and 56 mg/kg [2]. This study evaluated the single oral administration of fluralaner in a chewable tablet at 3 times the highest expected clinical dose (168 mg fluralaner/kg body weight). The individual dose for each treated dog was based on the body weight determined one day before treatment. The tablet formulation used was the final commercial formulation intended to be marketed as Bravecto^™^, produced under Good Manufacturing Practice (GMP). Homogeneous distribution of fluralaner in tablets was previously confirmed as part of the product development. Whole fluralaner tablets (1400 mg fluralaner/tablet) and tablet portions were administered to each dog to deliver the calculated dose. The administration of cut tablets resulted in a maximum deviation from the target dose of 0.5 mg fluralaner/kg body weight. Following tablet administration, a small amount of water was administered to encourage swallowing. Control dogs remained untreated and were sham dosed with water only. On the treatment day (day 0), dogs of both groups were fed a portion of canned diet within 30 minutes prior to treatment and another portion of canned diet directly after treatment (approximately 350 grams in total). Dogs were fed around the time of treatment to ensure high systemic fluralaner exposure, since fluralaner bioavailability is higher in fed dogs [13]. The normal daily ration of food was offered one hour following treatment.

All dogs were observed by a technician for general health before treatment and during the first hour following fluralaner administration. Clinical assessments were performed by a veterinarian, who was masked to the treatment status of each dog, at 3, 6, 9, 12, 18, 24, 30, 36, 42, 48, 54, 60 and 72 hours after administration. Assessments focused on, but were not limited to: behaviour, salivation, vomiting, ataxia, muscular tremor, mydriasis and pupillary reflex. Between 3 and 7 days after treatment dogs were observed four times daily and then twice daily for the remaining 21 days of the study period by a technician masked to the treatment status of each dog. Additional physical examinations were performed by a veterinarian (masked to the treatment status) before fluralaner administration and on days 7, 14 and 28. The veterinary study director assessed all parameters recorded and all clinical findings for their relationship to fluralaner treatment. Any treatment-related findings were classified as adverse events. Blood samples were collected at intervals over the study period to monitor systemic exposure to fluralaner. Blood sampling time points were selected based on previous pharmacokinetic data [14] and blood samples were analyzed using a validated LC-MS/MS method (lower limit of quantification = 10 ng/ml).

## Results and discussion

All fluralaner-treated dogs rapidly consumed the complete portion of food offered prior to and directly after fluralaner administration.

**Figure 1:**
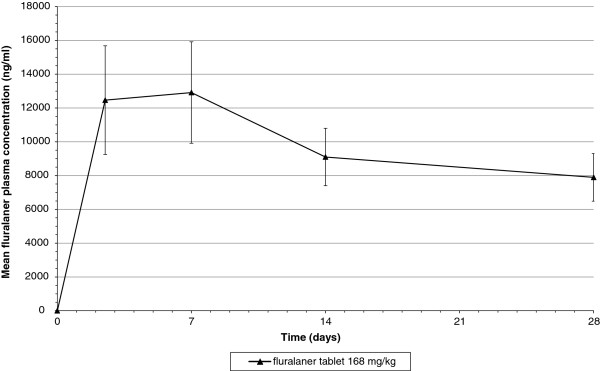
Mean fluralaner plasma concentration (± standard deviation) following oral administration at 168 mg/kg to MDR1(-/-) Collies.

Fluralaner was quantifiable in plasma of treated dogs from the first post-treatment sampling time point throughout the study, confirming dose-related systemic exposure (Figure 1).

No vomiting, excessive salivation or any other findings were observed during the first hour of clinical observation following treatment or during the frequent clinical assessments performed over the 3 days following fluralaner administration. Clinical findings observed over the study period included an abraded callus on the medial hock of one dog pre-treatment, a dog who bit its tongue during feeding pre-treatment, evidence of estrus in three dogs of the fluralaner group and an observation of tachycardia in a control-group dog. These clinical findings were minor and not related to fluralaner treatment.

This clinical safety evaluation of fluralaner, a novel systemic antiparasitic drug, in MDR1(-/-) dogs treated orally at 3 times the highest recommended clinical dose did not detect any adverse events. The detailed clinical observations were timed to provide maximum coverage during the period of highest expected systemic fluralaner concentrations to assure that potential neurological clinical signs would have become apparent. However, no signs of neurotoxicity, or any other adverse events, were observed during these frequent observations.

These results are consistent with a previous safety study [12] in healthy Beagle dogs, that found no evidence of neurotoxic symptoms or any other treatment-related findings associated with repeated oral administration of up to 280 mg fluralaner/kg.

## Conclusions

Fluralaner chewable tablets are well tolerated in MDR1(-/-) Collies following oral administration.

## Competing interests

FMW, MJA, RKAR and MCN are employees of Merck / MSD Animal Health. AJP is employee of the University of Illinois.

## Authors’ contributions

FMW, AJP, MJA, RKAR and MCN authored the study design, monitored the study and interpreted the results. All authors revised and approved the final version of the manuscript.
